# Associations between ankle-brachial index, diabetes, and sleep apnea in the Hispanic community health study/study of Latinos (HCHS/SOL) database

**DOI:** 10.1186/s12872-020-01402-7

**Published:** 2020-03-05

**Authors:** Mohammed M. Alshehri, Abdulfattah S. Alqahtani, Aqeel M. Alenazi, Monira Aldhahi, Shaima Alothman, Corey Gray, Bader Alqahtani, Kamlesh Khunti, Patricia Kluding

**Affiliations:** 1 Department of Physical Therapy and Rehabilitation Science, University of Kansas Medical Center, 3901 Rainbow Blvd, MS 2002, Kansas City, KS 66160 USA; 2 Physical Therapy department, Jazan University, Jizan, Saudi Arabia; 3 Department of Health Rehabilitation Sciences, College of Applied Medical Sciences, King Saud University, Riyadh, Kingdom of Saudi Arabia; 4 Department of Physical Therapy and Rehabilitation Science, Prince Sattam bin Abdulaziz University, Alkharj, Saudi Arabia; 5 Department of Rehabilitation Sciences, College of Health and Rehabilitation Sciences, Princess Nourah Bint Abdulrahman University, Riyadh, Kingdom of Saudi Arabia; 6 Diabetes Research Centre, University of Leicester, Leicester, UK

**Keywords:** Diabetes, Ankle-brachial index, Obstructive sleep apnea, Apnea-hypopnea index

## Abstract

**Background:**

Sleep apnea and diabetes mellitus (DM) negatively impact cardiovascular health. One important indicator of cardiovascular health is the Ankle-Brachial Index (ABI). Either low ABI or high ABI are signs of peripheral vascular impairment. Impaired vascular health and DM, together, might provoke sleep apnea; however, information regarding these relationships is limited. Therefore, this study aimed to investigate the association between ABI, DM status, and severity of obstructive sleep apnea in people of Hispanic/Latino descent who are diverse in culture, environmental exposures, nativity, socioeconomic status, and disease burden.

**Methods:**

A cross sectional analysis from a multi-center epidemiologic study, Hispanic Community Health Study/Study of Latinos, was utilized and included 3779 participants (mean age 55.32 ± 7.67, females 57.9%). The sample was divided into 4 groups based on the American Diabetes Association diagnostic guidelines (no DM or DM), and the ABI status (normal and abnormal). Multiple linear regression analysis was used to determine the association of the four groups and other independent variables with severity of sleep apnea measured by apnea-hypopnea index. Kruskal-Wallis H test was used for comparisons between groups for the apnea-hypopnea index. The significant level was set at 0.01.

**Results:**

There were significant differences between groups in the mean of apnea-hypopnea index (*P* < 0.001; no DM + normal ABI = 5.42 ± 9.66, no DM + abnormal ABI = 7.11 ± 11.63, DM + normal ABI = 10.99 ± 15.16, DM + abnormal ABI = 12.81 ± 17.80). Linear regression showed that DM and abnormal ABI were significantly associated with severe sleep apnea (β = 3.25, *P* = 0.001) after controlling for age, sex, BMI, income, education, alcohol use, cigarette use, hypertension or related medication, stroke and statin use.

**Conclusion:**

These findings suggest that people with DM and abnormal ABI were more likely to have high apnea-hypopnea index compared to the other groups. We observed gradual increasing in the severity of sleep apnea from low abnormality groups to high abnormality groups for Hispanic/Latino. Further work should elucidate the association of DM, abnormal ABI and sleep apnea with longer term outcomes, and replicate this work in different populations.

## Background

Diabetes mellitus (DM) is an evolving medical problem that has taken a serious toll on the physical health condition leading to cardiovascular and metabolic disorders [1, 2]. The worldwide burden of the DM is projected to escalate by 2030 to over 450 million people [3]. DM is associated with both microvascular and macrovascular complications [4]. The presence of DM is an important contributor for predisposing patients to the development and progression of peripheral artery disease (PAD) [5]. The epidemiological evidence has shown that an abnormal ankle-brachial index (ABI) is prevalent in 20% of people with impaired glucose intolerance while ABI is prevalent in just 7% of those with normal glucose tolerance [5]. The relationship between PAD and DM is noted to be reciprocal and these combined comorbidities may lead to impaired functional status. The coexistence of the PAD in individuals with DM have exacerbated by physiological stressor such as episodic bout of sleep apnea, which is known to accentuate atherogenic risk due to upregulation of prothrombotic markers and biomarkers of systemic inflammation [1, 2].

Obstructive sleep apnea (OSA) is common and affects 3–9% of women and 10–17% of men between the ages of 30 years and 70 years [6]. OSA is a nocturnal physiologic stressor which disturbs the body homeostasis and results in episodes of complete or partial airway obstruction that occur during sleep which interfere with the normal physiology of sleep-heart interaction and associated with thoracoabdominal effort [6]. In contrast, central sleep apnea results from loss of ventilatory drive and induces cessation of breathing with no thoracoabdominal effort and most commonly occurs in patients with heart failure [7]. A number of health-related conditions are associated with OSA, including hypertension and increased risk of cardiovascular disease [8]. OSA may have an additive, adverse impact on insulin sensitivity, due to the mechanism of sympathetic activation, as well as alterations in growth hormone and cortisol secretion [9]. These effects may result in significant social or work-related difficulties and compromise quality of life and daytime functioning of patients with OSA [10]. However, the association of OSA in patients with coexistent diabetes and PAD remain to be elucidated.

Understanding the health status, cardiovascular diseases, epidemiology of different comorbidities in US Hispanics/Latinos is needed. The associations between SA, DM and PAD are poorly understood in US Hispanic/Latinos. This population is the largest minority in the US which constitutes 17% of the total US population [11]. This population have diversity in culture, environmental exposures, nativity, socioeconomic status, and disease burden, which might reflect differently to other populations [12, 13]. The epidemiological study in sleep traits and cardiovascular risk among the U.S. Hispanic/Latino population highlights a high prevalence of OSA [14]. The high prevalence of the OSA among this population predispose them to high risk of cardiovascular disease [15, 16]. Limited progress has been made to understand the bidirectional link between the impaired vascular health, DM, and severity of OSA. The information concerning these relationships has not been fully established. Therefore, this study aimed to elucidate the association between ABI, DM status, and severity of sleep apnea in people of Hispanic/Latino descent.

## Methods

### Study design and participants

For the present cross-sectional study, we analyzed data from the Hispanic Community Health Study/Study of Latinos (HCHS/SOL) from February 2010 to June 2011. The HCHS/SOL is a multisite community-based cohort study that examines the chronic diseases prevalence and the associated health factors in a sample of Hispanic/Latino adults [17]. Overall 14,109 Hispanic/Latino adults aged from 18 to 74 years participated in this study. Participants were recruited from randomly selected households in four U.S. field centers including Bronx, NY; Chicago, IL; Miami, FL; and San Diego, CA. Each site followed the same protocol of HCHS/SOL to collect baseline data. The HCHS/SOL adhered to the Declaration of Helsinki and all obtained data was approved by the institutional review boards at each field center. All participants were consented by the study’s reading and coordinating centers, and the methods and objectives of the study have been published previously [17, 18].

**Fig. 1:**
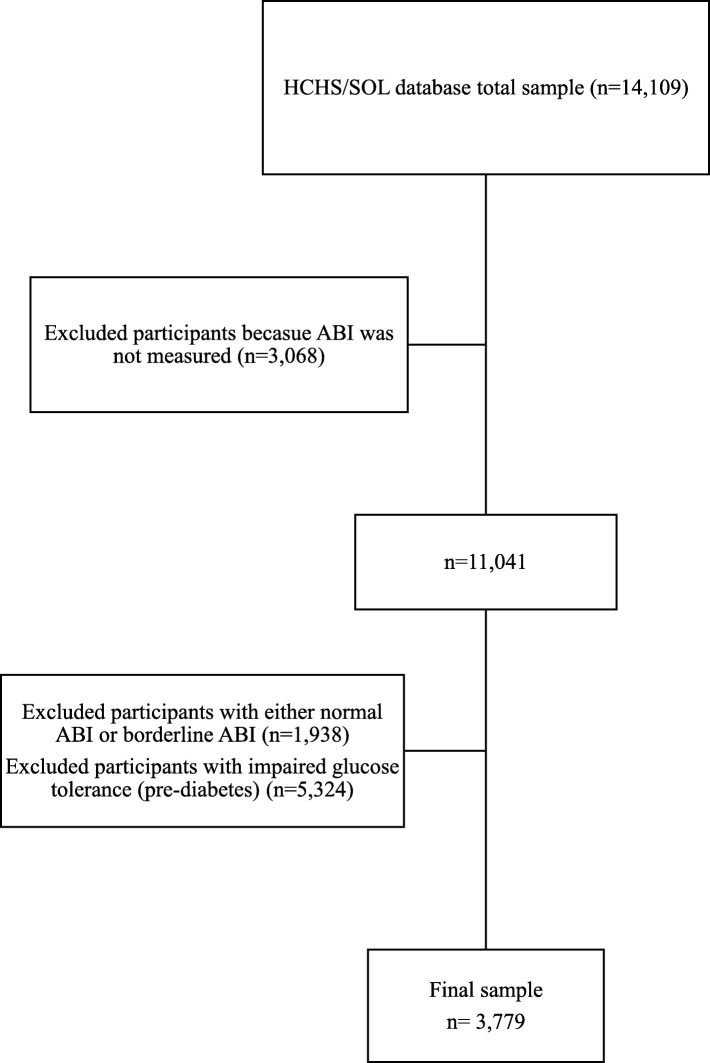
HCHS/SOL: Hispanic community health study/study of Latinos; ABI: Ankle-Brachial Index

The flowchart of this study is provided in Fig. 1. We excluded 3068 participants as their ABI was not measured. In addition, a total of 1938 participants with either normal ABI or borderline ABI, and 5324 participants with impaired glucose tolerance (pre-diabetes) were excluded from the analysis. Our final sample size was 3779 participants with an age range between 20 and 75 years. Baseline clinical and anthropometric characteristics assessments were obtained including self-reported questionnaires, blood labs, anthropometry, cardiovascular assessments, and home-based sleep monitor.

### Group classification

A total of 3779 participants were divided into 4 groups based on American Diabetes Association (ADA) guidelines 2010 (no DM or DM), and ABI categories (normal ABI or abnormal ABI. ADA guidelines define DM using fasting glucose level, oral glucose tolerance testing, hemoglobin A1c levels, and anti-diabetic medication use [19]. Based on the fasting time, DM was diagnosed if a fasting glucose value ≥126 mg/dl while fasting time was > 8 h, or if fasting glucose ≥200 while fasting time was < 8 h. In addition, DM was defined if glucose level ≥ 200 mg/dl 2 h after oral glucose ingestion, or hemoglobin A1c ≥6.5% or the use of antidiabetic medication. ABI was calculated as a mean of (systolic pressure at ankle/systolic pressure at arm). ABI was considered as abnormal ABI if overall ABI was ≥1.40 or ≤ 0.90, and ABI was considered normal ABI if the index was > 1.0 and < 1.4. The sample was classified as following: normal ABI and no DM group; normal ABI and DM group; ABI and no DM group; and abnormal ABI and DM group.

### Obstructive sleep apnea outcomes

Severity of OSA was measured objectively by overnight home-based objective testing with the Apnea Risk Evaluation System (ARES) (Unicorder 5.2; B-Alert, Carlsbad, CA) [20]. The ARES Unicorder (Advanced Brain Monitoring) acquires data on hemoglobin oxygen saturation, pulse rate (reflectance oximetry), snoring level (microphone), and head position/movement (actigraphy) were used to monitor overnight sleep activity. Decline of the airflow identified a respiratory event by 50% or higher for a period of ≥10 s. The desaturation level was measured by (SpO2) as the primary signal, and an insight software analyzed changes in pulse rate, snoring sounds, head movement, and the slope of the resaturation curve to identify behavioral markers of arousal that follow desaturations events.

A common measure for OSA severity is apnea-hypopnea index that is widely used in sleep studies [21]. The apnea-hypopnea index is the number of respiratory events per estimated sleep hour with considering desaturation events 0, 1, and 3%. The American Academy of Sleep Medicine criteria of apnea-hypopnea index 3% desaturation event was selected due to the good sensitivity and specificity with measures attained by polysomnography [22]. ARES data was scored by a certified polysomnologist from a sleep center.

### Data analysis and statistical plan

For the descriptive purpose, the data were presented as means with standard deviations (SD) for continuous variables and counts with percentages for categorical variables. Differences between the four groups were analyzed using Pearson chi-square for categorical variables or one-way analysis of variance for continuous variables. Kruskal-Wallis H test was used for comparisons between groups for the apnea-hypopnea index. Multiple linear regression analyses were used to investigate the association of DM status (no DM or DM) and ABI (normal ABI versus abnormal ABI) with apnea-hypopnea index. Reference category for predictor variable (groups) was set as no DM and normal ABI. We controlled for 4 sociodemographic variables including age as a continuous variable, gender (male or female), educational level (less than high school; high school or equivalent degree; or more than high school), and income (< $10,000; $10,000–$20,000; $20,001–$40,000; $40,001–$75,000; or More than $75,000). In addition, when modeling the association, we also controlled for cigarette use and alcohol use, which were categorized into 3 categories (Never, former, or current). We also included covariates capturing hypertension which was defined as a systolic blood pressure ≥ 140 and/or diastolic blood pressure ≥ 90 mmHg, or self-reported use of antihypertensive medications. Prevalent stroke or Traumatic brain injury and statin use were classified as (0 = not prevalent; 1 = prevalent, and 0 = no; 1 = yes, respectively). Body mass index (BMI) was calculated by measuring weight and height and controlled as a continuous variable.

Two models were created with groups as a predictor and apnea-hypopnea index as the dependent variable. Model 1 was adjusted for age and sex, and model 2 was adjusted for age, sex, BMI, income, education, alcohol use, cigarette use, hypertension or related medication, stroke and statin use. These covariates were selected due to their well-known associations with OSA or ABI. Results presented as Beta coefficient (β) with their standard error and *P*-values. Beta indicates an increase or decrease in the apnea-hypopnea index for each group to the reference group after controlling for other covariates. All statistical analyses were performed using Statistical Package for the Social Sciences for Macintosh, version 25.0 (SPSS Inc., Chicago, IL). Alpha level was set at 0.01.

## Results

A total of 3779 were included in this study. The participants were classified into four groups based on the presence or absence of DM and the category of ABI as either normal or abnormal. These groups included: participants without DM who had normal ABI (*n* = 1724; 45.62%), participants without DM who had abnormal ABI (*n* = 112; 2.96%), participants with DM who had normal ABI (*n* = 1659; 43.9%) and participants with DM who had abnormal ABI (*n* = 284: 7.52%). Table 1 shows participants’ characteristics of all enrolled subjects. There were statistically significant differences between groups in terms of age, sex, BMI, education, income, alcohol use, cigarette use, hypertension, stroke and statin use. Participants with DM and abnormal ABI showed the highest apnea-hypopnea index (12.83 ± 17.8, *p* < 0.001) compared to those in other groups.

**Table 1:** Physical and demographic characteristics of all enrolled subjects

Clinical/ physical features	Total sample ***n*** = 3779	No DM and normal ABI Group ***n*** = 1724	No DM and abnormal ABI Group ***n*** = 112	DM and normal ABI Group ***n*** = 1659	DM and abnormal ABI Group ***n*** = 284	***p-***value
**Age, years (mean ± SD)** ***n*** **= 3779**	55.32 ± 7.66	52.81 ± 6.63	55.13 ± 7.79	57.12 ± 7.71	60.14 ± 7.96	< 0.001
**Gender, Female,** **n (%)**	2187 (57.9)	1007 (58.4)	64 (57.1)	959 (57.8)	157 (55.3)	0.80
**BMI (mean ± SD)** ***n*** **= 3774**	30.05 ± 5.74	28.21 ± 4.65	27.69 ± 5.83	31.85 ± 6.01	31.68 ± 6.19	< 0.001
**Education, n (%)** ***n*** **= 3493**						< 0.001
**Less than HS**	1610 (42.9)	607 (35.3)	40 (35.7)	817 (49.8)	146 (52)	
**HS or equivalent**	809 (21.6)	397 (23.1)	24 (21.4)	338 (20.6)	50 (17.8)	
**> HS or equivalent**	1335 (35.6)	715 (41.6)	48 (42.9)	487 (29.7)	85 (30.2)	
**Income, n (%)** ***n*** **= 3442**						0 < .001
**< $10,000**	610 (17.7)	244 (15.2)	24 (24.5)	275 (18.4)	67 (27.2)	
**$10,001-20,000**	1154 (33.5)	513 (32.0)	30 (30.6)	529 (35.4)	82 (33.3)	
**$20,001-40,000**	1124 (32.7)	549 (34.2)	36 (36.7)	479 (32.0)	60 (24.4)	
**$40,001-75,000**	402 (11.7)	213 (13.3)	5 (5.1)	155 (10.4)	29 (11.8)	
**> $75,000**	152 (4.4)	84 (5.2)	3 (3.1)	57 (3.8)	8 (3.3)	
**Alcohol use, n (%)** ***n*** **= 3498**						< 0.001
**Never**	781 (20.8)	320 (18.6)	25 (22.3)	366 (22.2)	70 (24.9)	
**Former**	1402 (37.3)	537 (33.3)	36 (32.1)	677 (41.1)	116 (41.3)	
**Current**	1576 (41.9)	827 (48.1)	51 (45.5)	603 (36.6)	95 (33.8)	
**Cigarette use, n (%)** ***n*** **= 3494**						< 0.001
**Never**	2071 (55.2)	935 (54.4)	51 (45.9)	944 (57.4)	141 (50.2)	
**Former**	975 (26)	408 (23.7)	26 (23.4)	458 (27.9)	83 (29.5)	
**Current**	708 (18.9)	375 (21.8)	34 (30.6)	242 (14.7)	57 (20.3)	
**Hypertension, n (%)** ***n*** **= 3516**						< 0.001
**Yes**	1624 (43.0)	403 (23.4)	45 (40.2)	974 (58.7)	202 (71.1)	
**Stroke, n (%)** ***n*** **= 3499**						< 0.001
**Yes**	146 (3.9)	42 (2.4)	4 (3.6)	78 (4.7)	22 (7.9)	
**Statin use, n (%)** ***n*** **= 3437**						
**Yes (%)**	809 (21.9)	122 (7.3)	11 (10.1)	567 (34.8)	109 (39.5)	< 0.001
apnea-hypopnea index ***n*** **= 3340**	8.46 ± 13.38	5.42 ± 9.66	7.11 ± 11.63	10.99 ± 15.16	12.81 ± 17.80	< 0.001

Values are presented as means ± SD or n (%). A Pearson chi-square test or one-way analysis of variance were used to determine *p*-values between all groups

Abbreviations: *DM* diabetes, *ABI* ankle brachial index, *BMI* body mass index, *HS* high school, *$* United States dollar

Table 2 shows the results of linear regression. Model 1 showed that having DM and normal ABI was associated with approximately 4 score increase in apnea-hypopnea index (β = 4.82, *P* < 0.001), and having DM and abnormal ABI was associated with about 6 score increase in apnea-hypopnea index (β = 6.17, *P* < 0.001) after controlling for age and gender. However, there was no association with apnea-hypopnea index in participants without DM who had abnormal ABI in both models. Model 2 showed that participants with DM and normal ABI had not significant score in apnea-hypopnea index (β = 1.45, *P* = 0.018), whereas individuals with DM and abnormal ABI had a significant association with a high score of apnea-hypopnea index (β = 3.25, *P* = 0.001) after controlling for all covariates.

**Table 2:** Multiple linear regression analyses for the association between DM, ABI and apnea-hypopnea index

Predictor Variable	Model 1 (***n*** = 3114)			Model 2 (***n*** = 2797)		
	β	SE	***p***-value	β	SE	***p-value***
**No DM and normal ABI**	Ref	–	–	–	–	–
**No DM and abnormal ABI**	1.38	1.34	0.30	1.74	1.45	0.23
**DM and normal ABI**	4.82	0.48	< 0.001	1.28	0.54	0.018
**DM and abnormal ABI**	6.17	0.90	< 0.001	3.25	0.98	0.001

Model 1, adjusted for age and gender; Model 2, adjusted for model 1 in addition to body mass index, income, educational level, alcohol use, cigarette use, hypertension, stroke and statin use; ref., this group (No DM and normal ABI) was set as reference category

Abbreviations: *DM* diabetes, *ABI* ankle brachial index, *β* Beta coefficient, *SE* standard error

## Discussion

The findings in this study highlighted the association between abnormal ABI and DM with increasing apnea-hypopnea index. These findings are in agreement with previous studies that examined this association with either PAD or DM. To our knowledge, this is the first study to examine the multimorbidity of PAD and DM as associated risk factor on severity of OSA. By understanding the association between ABI, DM status, and severity of sleep apnea, health care providers can appreciate the public health burden of OSA and offer insights into the therapeutic avenues available for these patients.

Our results showed that Hispanic/Latino population with abnormal ABI and DM are more likely to have increased 6 score points on the apnea-hypopnea index compared to those without DM and normal ABI groups. A substantial body of evidence has established that the OSA and DM share several risk factors [8] and have bidirectional relationship [23]. Also, they are associated with the risk of developing cardiovascular diseases such as PAD. One study assessed the arterial stiffness in 71 participants with Type 2 DM with and without OSA using pulse wave velocity [24]. Although their sample were older adults with high risk factors of cardiovascular disease including high BMI and systolic blood pressure, the study showed no association between OSA and arterial stiffness [24]. In contrast with what was previously thought [24], our findings might be due to different methodological procedures and possibility unknown diagnosis of comorbidities in the comparison group. However, there is a need to understand the underlying mechanisms of this combination, PAD and DM, on OSA severity.

The study results showed that SA was not associated with PAD in people without DM. This result is in contrast with several studies assessed the association between OSA and PAD. For example, a study utilizing the same cohort showed that moderate-to-severe OSA is associated with higher odds of PAD [25]. However, they only controlled for DM without taking in account pre-diabetes. The different in results might be due to that we excluded individuals with pre-diabetes. Similarly, another study examined the relationship of SA and PAD in 59 individuals found that OSA is prevalent in those people. However, this relationship seems to be mediated by the presence of DM [26]. Overall, future population cohort might want to investigate in depth the relationship between OSA and PAD in the presence and absent of DM.

Additionally, our study captures that the co-existence of all three co-morbidities (OSA, PAD, and DM) was associated with cardiovascular risk factors (high BMI and blood pressure) and further research needs to determine the co-existence of all three conditions with longer-term health outcomes. Considering a high technical failure rate of home sleep monitor, our results are still similar to previous studies that used polysomnography on either people with PAD or DM. Schaefer et al. showed increasing in brachial-ankle pulse wave velocity from people without OSA (apnea-hypopnea index < 5/h) to severe OSA (apnea-hypopnea index ≥30/h) [14]. In another population-based study, our findings were in line with the results that the severity of OSA had a significant effect and association with impaired fasting glucose levels after adjusting for demographics and sleep duration [27]. Further, Siwasaranond et al. observed that individuals with moderate-to-severe OSA and DM were 3.05 times more likely to experience DM-related complications, such as neuropathy and coronary artery disease [28]. In another study, PAD was found to be significantly associated with SA, however, the association was attenuated after adjusting for multiple comorbidities, including DM. This indicates that DM along with other comorbidities plays an important role in OSA severity [29].

In this study, we attempted to control for common comorbidities and risk factors associated with PAD, DM, and OSA [30]. In addition, we controlled for sociodemographic variables that might influence sleep quality or PAD and DM management. Similar to global estimate the results of this study indicated that PAD was more prevalent in older individuals with hypertension and dyslipidemia [31]. PAD globally has a higher prevalence in women compared in men [32, 33]. However, in high income countries, PAD has been reported to be more prevalent in men compared to women which is consistent with our results [32]. Our results showed that BMI, as expected, was higher in people with DM in agreement with the global estimate [34]. Furthermore, our results indicated an additive effect of PAD and DM presence on the percentage of individuals with hypertension, stroke, and dyslipidemia. To our knowledge, no studies examined this association between the three comorbidities in any populations, we recommend replicating this study to different populations. Hispanics/Latinos have diversity in genetics, lifestyle, culture, environment, and education, which might lead us to have different explanations toward this association.

In this study, presence of DM and abnormal ABI was associated with increased severity of OSA. Our findings are not directly comparable to other studies, as the association of both DM and abnormal ABI has not been studied together in relation to OSA. However, when looking to them separately, previous studies reported an association between DM and SA [35], and a strong association between abnormal ABI and SA in different populations [25, 36]. This suggests that decreased arterial compliance, as resultant of PAD in patients with pre-existence diabetic may contribute to a vicious pathophysiological cycle revolving around OSA. DM has been shown to lead to PAD by way of metabolic changes as well as microdamage to the inner linings of arteries which can cause atherosclerosis and thus restrict oxygenated blood flow to the peripheral arteries such as the carotid artery [37]. Furthermore, OSA itself can contribute to its own severity exacerbation through increases in sympathetic neural activity resultant from OSA-related hypoxic events [38, 39]. Chronic basal sympathetic overactivity is a common observation in people with OSA as well as in people with DM and can lead to damage of the endothelial walls of the vasculature and thus limiting blood flow, as seen in PAD [40]. It should also be noted that impaired vagal function could limit anti-inflammatory production and thus contribute to atherosclerosis [40, 41].

The study has several potential limitations. First, the current findings were drawn from cross-sectional data, which limits the ability to make causal inferences. Future studies using longitudinal data would help to understand the causality of the relationship between DM, PAD, and sleep apnea. Second, the present results may not be generalizable to all US Hispanics/Latinos or general populations, since the current sample of the HCHS/SOL study was collected from only four US cities. Third, although the tools used in the study are sensitive for apnea-hypopnea index and ABI measures, there is a need to confirm our findings using gold standard tools such as polysomnography and pulse wave velocity. Fourth, we were unable to differentiate between type 1 and type 2 DM. However, based on ADA 2013, the prevalence of type 2 DM is higher than type 1 DM which limits the effect of this imprecision on our findings. Finally, we were unable to identify continuous positive airway pressure user originally versus not, which might have a carryover effect.

## Conclusion

The findings of the current study provide evidence of the association between DM, PAD, and severity of OSA. People with DM and abnormal ABI were more likely to have increased apnea-hypopnea index compared to the other groups. Furthermore, the established relationship in our study emphasizes the importance of identifying and treating people with PAD and DM to prevent or reduce severity of OSA. Further work should elucidate the association of DM, abnormal ABI and OSA with longer term outcomes.

## Data Availability

The HCHS/SOL was carried out as a collaborative study supported by contracts from the NHLBI to the University of North Carolina (N01-HC65233), University of Miami (N01- HC65234), Albert Einstein College of Medicine (N01-HC65235), Northwestern University (N01-HC65236), and San Diego State University (N01-HC65237). The HCHS/SOL project website for more information regarding accessing data is http://www.cscc.unc.edu/hchs/ [43]. The data for the current study are available from HCHS/SOL on https://sleepdata.org/datasets/hchs [44]. However, restrictions apply to the availability of these data, and are not publicly available. MMA received administrative permission to access and use the sleep data. The following institutes/centers/offices contributed to the baseline HCHS/ SOL funding period through a transfer of funds to the NHLBI: National Institute on Minority Health and Health Disparities, National Institute on Deafness and Other Communication Disorders, National Institute of Dental and Craniofacial Research, National Institute of Diabetes and Digestive and Kidney Diseases, National Institute of Neurological Disorders and Stroke, and National Institutes of Health Office of Dietary Supplements.

## Abbreviations

ABI: Ankle-brachial index
ADA: American Diabetes Association
ARES: Apnea risk evaluation system
BMI: Body mass index
DM: Diabetes mellitus
HCHS/SOL: Hispanic community health study/study of Latinos study
OSA: Obstructive sleep apnea
PAD: Peripheral artery disease
